# Co-fermentation Involving *Saccharomyces cerevisiae* and *Lactobacillus* Species Tolerant to Brewing-Related Stress Factors for Controlled and Rapid Production of Sour Beer

**DOI:** 10.3389/fmicb.2020.00279

**Published:** 2020-02-21

**Authors:** Anna Dysvik, Sabina Leanti La Rosa, Kristian Hovde Liland, Kristine S. Myhrer, Hilde Marit Østlie, Gert De Rouck, Elling-Olav Rukke, Bjørge Westereng, Trude Wicklund

**Affiliations:** ^1^Faculty of Chemistry, Biotechnology and Food Science, Norwegian University of Life Sciences, Ås, Norway; ^2^Faculty of Science and Technology, Norwegian University of Life Sciences, Ås, Norway; ^3^Norwegian Institute of Food, Fisheries and Aquaculture Research, Ås, Norway; ^4^Faculty of Engineering Technology, KU Leuven, Ghent, Belgium

**Keywords:** sour beer, *Lactobacillus*, *Saccharomyces cerevisiae*, sensory analysis, mixed fermentation, lactic acid, alcoholic beverage

## Abstract

Increasing popularity of sour beer urges the development of novel solutions for controlled fermentations both for fast acidification and consistency in product flavor and quality. One possible approach is the use of *Saccharomyces cerevisiae* in co-fermentation with *Lactobacillus* species, which produce lactic acid as a major end-product of carbohydrate catabolism. The ability of lactobacilli to ferment beer is determined by their capacity to sustain brewing-related stresses, including hop iso-α acids, low pH and ethanol. Here, we evaluated the tolerance of *Lactobacillus brevis* BSO464 and *Lactobacillus buchneri* CD034 to beer conditions and different fermentation strategies as well as their use in the brewing process in mixed fermentation with a brewer’s yeast, *S. cerevisiae* US-05. Results were compared with those obtained with a commercial *Lactobacillus plantarum* (WildBrew^TM^ Sour Pitch), a strain commonly used for kettle souring. In pure cultures, the three strains showed varying susceptibility to stresses, with *L. brevis* being the most resistant and *L. plantarum* displaying the lowest stress tolerance. When in co-fermentation with *S. cerevisiae*, both *L. plantarum* and *L. brevis* were able to generate sour beer in as little as 21 days, and their presence positively influenced the composition of flavor-active compounds. Both sour beers were sensorially different from each other and from a reference beer fermented by *S. cerevisiae* alone. While the beer produced with *L. plantarum* had an increased intensity in fruity odor and dried fruit odor, the *L. brevis* beer had a higher total flavor intensity, acidic taste and astringency. Remarkably, the beer generated with *L. brevis* was perceived as comparable to a commercial sour beer in multiple sensory attributes. Taken together, this study demonstrates the feasibility of using *L. brevis* BSO464 and *L. plantarum* in co-fermentation with *S. cerevisiae* for controlled sour beer production with shortened production time.

## Introduction

Lactic acid bacteria (LAB) are gram-positive, non-sporulating bacteria with lactic acid as their main product of carbohydrate metabolism (Zhu et al., 2010). LAB include homofermentative members, converting hexose sugars almost exclusively to lactic acid, and heterofermentative species fermenting hexose sugars to lactic acid, CO_2_ and ethanol or acetic acid (Von Wright and Axelsson, 2019). *Lactobacillus* is a genus within the LAB group with metabolism that is either obligate homofermentative (e.g. *L. acidophilus* and *L. delbrueckii*), obligate heterofermentative (*L. brevis* and *L. buchneri*) or facultative heterofermentative (*L. plantarum* and *L. sakei*) (Ibrahim and Ouwehand, 2019). Lactobacilli are frequently associated with food and beverages produced through mixed fermentations where both bacteria and yeast are involved. Examples of such products include wine (Mtshali et al., 2012; Wang et al., 2018), kefir (Guzel-Seydim et al., 2011), sake (Tsuji et al., 2018), sour dough bread (Minervini et al., 2014; Ripari et al., 2016), and beer (Vriesekoop et al., 2012).

Beer with intentional acidic taste, referred to as sour beer, is traditionally fermented as a spontaneous process where wort is inoculated by environmental exposure rather than active microbial inoculation (Van Oevelen et al., 1977; Verachtert and Derdelinckx, 2014). The traditional production methods for sour beer, such as Lambic and Geuze beers, originate from Belgium and are still in use today. The complex, multi-microbial fermentations that progress through these methods entail huge time investments, up to 3 years, and are challenging to control (Van Oevelen et al., 1977; Verachtert and Derdelinckx, 2014). Due to the difficulties associated with traditional sour beer production, and the increasing popularity of sour beer in recent decades, alternative production methods are being explored (Peyer et al., 2017; Osburn et al., 2018; Alcine Chan et al., 2019). During sour beer fermentation, yeasts generate ethanol and other metabolic products in the same manner they would in ale or lager fermentations. The presence of acid producing bacteria results in beer products with higher content of organic acids and reduced pH compared to other non-sour beers (Van Oevelen et al., 1976, 1977). Lactic and acetic acid constitute the most pronounced contribution from lactobacilli to the sensory properties of sour beer; in addition, lactobacilli have been proven to produce a wide range of other flavor-important metabolic products, including aldehydes, alcohols and esters (Salmerón et al., 2015; Dongmo et al., 2016; Stefanovic et al., 2017). The production of flavor-active, metabolites by *Lactobacillus* is strain dependent (Cui et al., 2019).

During the process of fermentation, the environmental conditions in which microbes reside are highly dynamic. Nutrients are depleted, metabolites such as organic acids and ethanol are produced, and cell densities increase. The ability of microorganisms to quickly adapt to these conditions are vital for their survival and continued metabolism. Previous studies have shown that exposure to environmental stresses results in changed gene expression in *Lactobacillus* (Guchte et al., 2002), ultimately shifting the composition of the produced flavor-active metabolites and the organoleptic properties of the fermented food products (Serrazanetti et al., 2009). Stress induced shifts in production of metabolites from *Lactobacillus* sp. have been proven in different types of food, such as milk (Østlie et al., 2005), fruit and vegetables (Wu et al., 2015) and kefir fermentation.

Beer during and after fermentation represents a stressful environment for multiple purposes. Low pH, presence of ethanol, low oxygen, nutrient depletion and presence of anti-microbial hop compounds all contribute to making beer relatively stable regarding microbial infection (Vaughan et al., 2005). Some bacteria, however, can tolerate the harsh beer environment, which is unfortunate when their presence is unwanted (beer spoilers) but vital for production of sour beer. Lactobacilli are associated with both beer spoilage and delibirate sour beer fermentations (Vriesekoop et al., 2012). Even though these lactobacilli can grow in beer, the environmental factors influence their metabolism. Lactobacilli are known as relatively tolerant toward ethanol compared to other bacteria (Gold et al., 1992; G-Alegría et al., 2004), and relatively low concentrations of ethanol have even proven to stimulate the metabolism of certain LABs (Mateo et al., 2010).

Literature is scarce on the impact of environmental stress factors on metabolite production by lactobacilli in the beer environment (Peyer et al., 2017; Alcine Chan et al., 2019). Therefore, the objective of the current study was to investigate the effect of beer-related stress factors on growth and metabolite production by three different lactobacilli in wort medium. The selected strains were *Lactobacillus brevis* BSO464, a strain previously proven as resistant to the harsh beer environment (Bergsveinson et al., 2016); *Lactobacillus plantarum* (WildBrew^TM^ Sour Pitch), a commercial brewing strain commonly used for kettle souring (biologic acidification of wort prior to yeast fermentation); and *Lactobacillus buchneri* CD034, a strain previously used in research on kettle souring (Dysvik et al., 2019) but originally isolated from silage grass (Heinl et al., 2012). Controlled co-fermentations with lactobacilli and *Saccharomyces cerevisiae* were evaluated as a time-saving method for sour beer production. The produced beers were assessed with respect to degradation of carbohydrates and amino acids, production of flavor-active metabolites as well as sensory properties.

## Materials and Methods

### Yeast, Bacterial Strains and Growth Conditions

The three *Lactobacillus* strains used in this study were *Lactobacillus brevis* BSO464, purchased from Campden BRI (Gloucestershire, United Kingdom); *Lactobacillus plantarum* (WildBrew^TM^ Sour Pitch), purchased from Lallemand; and *Lactobacillus buchneri* CD034, kindly donated by the Department of Biotechnology at the University of Natural resources and Life Sciences, Vienna, Austria. Starter cultures for all three strains were prepared by propagating the bacteria twice in MRS medium (De Man, Rogosa and Sharpe, Merck, Darmstadt, Germany) and twice in wort medium at 30°C overnight. Cells were harvested by centrifugation (9000 × *g*, 10 min, 4°C), resuspended in wort medium (see below) supplemented with 15% glycerol (v/v) and stored at −80°C. The starter cultures were thawed at 4°C prior to use and inoculated directly. Viability after freezing and thawing was checked, and inoculations were made accordingly. The *Saccharomyces cerevisiae* US-05 was purchased from Fermentis (France). Dry yeast was rehydrated in sterile water at 1:10 (w/v) for 30 min at 22°C prior to inoculation in fermentation experiments. Unless otherwise stated, all fermentations were carried out in triplicate at 22°C under static conditions.

### Wort Production

The wort used for the stress experiments and small-scale co-fermentations was prepared by diluting concentrated brewer’s wort (Pilsen Light, Pure malt extract, Briess Malt and Ingredient Co, Chilton, WI, United States) in water at 120 g/L. The solution was autoclaved, solid material was removed after the solution had cooled down and the remaining clear solution is hereafter referred to as wort medium. The wort medium had a specific gravity of 1.033 (8.4°P). In the larger scale brewing experiment, wort was prepared using a 60L PRO pilot scale brewery vessel from CoEnCo (Oostkamp, Belgium, 2014). Crushed malt (33% wheat malt from Weyermann, Bamberg, Germany and 67% Pilsner malt from BestMalz, Heidelberg, Germany) was mashed in water at 0.25 kg/L according to the following scheme: 45 min at 65°C, 15 min at 72°C and 2 min at 78°C. The wort was separated from the spent grain and boiled for 60 min, yielding wort with specific gravity of 1.038 (9.6°P). Both the wort produced from concentrate and from malt, was prepared without hops. When iso-α acid concentration is specified, the concentration was obtained by addition of pre-isomerized hop extract (Hop-extract pre-isomerized, 6%, Browland, Belgium) prior to inoculation.

### Stress Experiments

Fermentation bottles (50 mL) were prepared with wort media with various adjustments according to different stressors. The *reference* condition was wort medium, at 0% (v/v) ethanol, 0 mg/L iso-α acids, pH 5, inoculated with *Lactobacillus* (10^6^ CFU/mL), incubated at 22°C for 7 days. The conditions for the *high inoculation* trial differed from the reference regarding inoculation with 10^8^ CFU/mL. The conditions for the *high temperature* trial were identical to the reference except incubation of the flasks at 30°C. Wort media was supplemented with 5% (v/v) ethanol for the *Ethanol* trial, and 5 mg/L of iso-α acids for the *Iso-*α *acid* trial. Two different medias were prepared with reduced initial pH, one where the initial pH was reduced from pH 5 to 4 with lactic acid [*Low pH (lactic acid)*] and one where the initial pH was reduced from pH 5 to 4 with hydrochloric acid [*Low pH (HCl)].* Finally, a *multi-stressor* trial was conducted with wort media containing 5% (v/v) ethanol, 5 mg/L iso-α acids and reduced initial pH adjusted with lactic acid. Sampling was done at 0, 4, 8, 12, 24, 32, 48, 72 h, and at 5 and 7 days. Growth was monitored on MRS agar (VWR Chemicals, Leuven, Belgium) and pH was monitored using a Sentron pH-meter with SI probe (Sentron, Netherlands). After the final sampling (7 days), remaining content in each fermentation flask was centrifuged at 7000 × *g*, 10 min, 4°C and the supernatants stored at −20°C prior to metabolite analysis.

### Small Scale Co-fermentations With LAB and Yeast

The co-fermentation with lactobacilli and yeast was assessed in flasks containing 400 mL wort medium supplemented with 5 mg/L iso-α acids. Flasks were inoculated simultaneously with 10^7^ cells/mL of lactobacilli and 10^6^ cells/mL of yeast before incubation at 22°C for 21 days. Lactobacilli were inoculated at a higher ratio to give the bacteria an initial advantage and promote their contribution to the fermentation. The population dynamic was monitored during the fermentation process at established intervals (0, 14, 48, 72 h and 4, 5, 7, 14, and 21 days); samples were plated both on MRS agar supplemented with 25 mg/L cycloheximide (Sigma-Aldrich, St. Louis, MO, United States) and Rose-Bengal Chloramphenicol agar (RBC, Oxoid, Basingstoke, United Kingdom), to be able to differentiate lactobacilli populations from *Saccharomyces* populations, respectively. pH was monitored as described above. After the final sampling, 50 mL from each fermentation flask was centrifuged (7000 × *g*, 10 min, 4°C) and the supernatant was stored at −20°C prior to metabolite analysis. The remaining content from each flask was used for ethanol analysis.

### Large Scale Co-fermentation Experiment With LAB and Yeast

Fermentation tanks (10 L) were prepared with PRO pilot scale brewery wort supplemented with 5 mg/L iso-α acids. Inoculations, fermentation temperature and duration and monitoring of population dynamic were carried out as described above. Samples (80 mL) were withdrawn throughout fermentation, centrifuged (7000 × *g*, 10 min, 4°C) and the supernatant was kept at −20°C for analysis of amino acids, carbohydrates, metabolites and ethanol. After the final sampling, tanks were kept at 4°C for 14 days before the beer was slightly carbonated using an Aqvia sodastreamer (AGA, Luleå, Sweeden) and transferred to 0.33 L bottles for sensory analysis. Beer fermentations were carried out in triplicate with yeast alone, yeast in co-fermentation with *L. brevis* and yeast in co-fermentation with *L. plantarum*.

### Headspace Gas Chromatography (HSGC)

Volatile compounds (types and corresponding IUPAC names can be revised in Supplementary Tables S2, S3) were detected and quantified by HSGC according to the method by Narvhus et al. (1998) with the following modifications. Samples (10.00 g) fermented by lactobacilli alone were directly transferred to headspace vials (Machery Nagel, Dueren, Germany), while samples (10.00 g) from co-fermentations by yeast and lactobacilli were first filtered through 602h 1/2 folding filters (pore size < 2 μm, Schleicher & Schuell, Dassel, Germany) to remove CO_2_. Teflon-coated septa with aluminum rings (PFTA/Si septa, Agilent Technologies, Wilmington, DE, United States) were used to seal the vials before they were placed in a 7679A automatic headspace sampler, with a headspace bath temperature of 50°C and manifold temperature of 60°C. The sampler was connected to a 6890 GC system with flame ionization detector (Agilent Technologies). Helium 6.0 (Aga, Norway) at low rate 5.0 mL/min was used as carrier gas. Samples were mixed (45 min, 70 shakes/min) prior to injection (0.5 min injection time, 10 psi pressure). Analytes were separated on a CP-SIL 5CB GC column (Varian, Middelburg, Netherlands) of 25 m × 0.53 mm I.D. with film thickness 5 μm. The system was operated by Open LAB EZChrom software (version A.04.05, Agilent Technologies) and identification and quantification were carried out according to calibration with external standards. The following temperature scheme was applied during analysis: 35°C for 5 min: increase of 10°C/min until 40°C and kept at 40°C for 2 min; increase of 30°C/min until 130°C and kept at 130°C for 4 min; increase of 30°C/min until 160°C and kept at 160°C for 4 min; increase of 10°C/min until 180°C and kept at 180°C for 2 min; increase of 10°C/min until 200°C and kept at 200°C for 2 min.

### High Performance Liquid Chromatography (HPLC)

Organic acids as well as fructose and maltotriose were detected and quantified by HPLC, according to the method described by Marsili et al. (1981) with the following modifications. Samples (1.00 g) were mixed with water (MilliQ), 0.5 M H_2_SO_4_ and acetonitrile in a MultiRS-60 BIOSAN turner (Montebello Diagnostics A/S, Oslo, Norway) operated at 30 rpm for 30 min. Samples were centrifuged for 15 min at 1470 × *g* using a Kubota 2010 centrifuge (Kubota Corporation, Tokyo, Japan) prior to filtration through 0.2 μm PTFE membrane (Acrodisc CR 13 mm Syringe Filter, PALL, United Kingdom). Organic acids were separated on an Aminex HPX-87H column (Bio-Rad Laboratories, Hercules, CA, United States) with 0.05 M H_2_SO_4_ as mobile phase and a flow rate of 0.4 mL/min. The column, operated at 30°C, was connected to a 1260 Infinity HPLC instrument (Agilent Technologies, Singapore) with pump, autosampler, column oven, RI-detector (refractive index, used for acetic acid, fructose and maltotriose) and diode array detector-ultra violet (DAD-UV) detector, used for the other organic acids. Openlab CDS software (Agilent Technologies) was used to operate the system and detection and quantification were done according to calibration with external standards. Maltose, sucrose and glucose were quantified by the K-MASUG enzymatic kit (Megazyme, Wicklow, Ireland), used according to the instructions.

### Statistical Analysis of Metabolic Products

Differences in metabolites from the stressor experiments were examined by Analysis of variance Simultaneous Component Analysis (ASCA) (Jansen et al., 2005). MATLAB (2019a, The Mathworks, Natick, MA, United States) was used to fit the ASCA model, which split the variation in the dataset in three according to strain, stressor and strain-stressor interaction related variation. Confidence ellipsoids (Liland et al., 2018) were used to display uncertainty of the effect level means in the ASCA scores, similarly to Tukey’s test in ANOVA. Uni-dimensional ANOVA for each compound was combined with Tukey’s test for honestly significant differences. The ANOVA with Tukey’s test was carried out using R 3.6.1 (R Core Team, 2019, Austria, Vienna), and statistical significance level was set at *p* < 0.05. Variation in metabolites from the small- and larger scale co-fermentations were analyzed by ASCA and ANOVA with Tukey’s test as described above.

### Ethanol and Apparent Degree of Fermentation (ADF)

Beer characterization was carried out using a PBA-B instrument (Anton Paar, Graz, Austria), consisting of a DMA 4500M density meter, an Alcolyzer Beer ME module, a CarboQC ME module and a PFD filling device. The instrument was operated through the Generation M software v2.42 (Anton Paar, Graz, Austria).

### Free Amino Acids

Free amino acids were identified and quantified using an HPLC method described by Bütikofer and Ardö (1999) and Moe et al. (2013) with the following modifications. Samples (5.00 g) were mixed with 5 mL internal standard solution (0.4 μmol/mL L-norvalin in 0.1 M HCl). The samples were mixed for 15 min (MultiRS-60 BIOSAN, Montebello Diagnostics AS, Oslo, Norway) before being placed for 30 min in an ultrasonic water bath (Brandson 2510, Soest, Netherlands). The samples were then centrifuged for 40 min at 4°C at 2500 × *g* (Thermo Scientific, Heraeus Multifuge X3R, Osterode, Germany) before the supernatant was mixed 1:1 with 4% trichloroacetic acid, kept on ice for 30 min and centrifuged for 5 min at 4°C at 15600 × *g* (Eppendorf 5415D Microcentrifuge, Eppendorf, Hamburg, Germany). The supernatant was filtered through 0.2 μm cellulose acetate filters (VWR, United States) and stored at −20°C prior to further preparation. Borate buffer (350 μL, 0.4 M, pH 10.2) was mixed with 50 μL samples, and the samples were derivatized by allowing 5 μL to react for 15 s with 5 μL *O*-phthaldialdehyde (OPA) solution prior to injection. The samples were analyzed using an Agilent 1200 HPLC system (Agilent Technologies, Singapore) consisting of a serial pump, auto injector, column oven, thermostat and fluorescence detector. The instrument was operated through Open LAB CDS software (Agilent Technologies). A sample volume of 10 μL was injected and analytes separated on an XTerra RP 18 column (150 × 4.6 mm; Waters, MA, United States) operated at 42°C. Two mobile phases were used at 0.7 mL/min: eluent A (30 mmol/L sodium acetate trihydrate, 0.1 mmol/L triplex III, 0.25% tetrahydrofuran, pH 7.2) and eluent B (100 mol/L sodium acetate trihydrate, 0.53 mol/L triplex III, 80% acetonitrile, pH 7.2). The derivatized amino acids were separated by a stepwise linear gradient from 3.3 to 20.7% eluent B over 13 min, from 20.7 to 30% eluent B over 12 min and from 30 to 100% eluent B over 4 min. Free amino acids in the samples were identified and quantified based on a standard curve generated with external standards.

### Sensory Evaluation of Produced Beers by Trained Panel

A professional sensory panel of eight trained assessors at the Norwegian Institute of Food, Fisheries, and Aquaculture Research (Nofima), Aas, Norway was used for the sensory evaluation of produced beers. All panelists were previously screened for sensory abilities (basic tastes, color vision, odor detection, tactile sensibility) and communication skills regarding sensory descriptions of products recommended in ISO 8586 (International Organisation for Standardisation [ISO], 2012) in a sensory laboratory designed in accordance with ISO 8589 (International Organisation for Standardisation [ISO], 2007). A list of 23 sensory attributes (Supplementary Table S1) was generated and agreed upon by the panel, based on a brain storming session and previous experiments with beer. The assessors were trained in the definition of the selected sensory attributes prior to the actual experiment. The three different beers were evaluated in duplicate in a Sensory profiling according to Generic Descriptive Analysis as described by Lawless and Heymann (2010). The samples were evaluated by each assessor within each session in individual randomized order. The evaluation of eight samples in total was conducted in four sessions. A warm-up sample served in the beginning of the first serving, and a commercial sour beer reference (Geuze, Mariage Parfait, 2015, Boon Brewery, Belgium) was evaluated in duplicate at the end of the last session. One bottle from each of the three replicates of the three different beers, were mixed in a beaker before serving. Two bottles of the commercial sour beer reference were mixed in a beaker. A decarbonation procedure was carried out by pouring the beer back and forth between bakers 20 times and leaving the beer to rest for 1 h prior to serving. The decarbonation of the commercial sour beer reference was done to obtain a similar carbonation level to the beers produced in this study. Clear plastic cups, tagged with random three-digit codes, were used to serve 30 mL of beer at 17 ± 1°C. All samples in one session were placed in the sensory evaluation booths at the same time and monadically evaluated at individual speed and registered continuously, using EyeQuestion (v4.11.33, Logic8, Holland). The assessors took a sip of the beer and rated all attributes by intensity on a non-structured continuous scale. The endpoints of this scale corresponded to 1 (lowest intensity) and 9 (highest intensity), and the scores were converted to a number between the endpoints by the Eye Question software. XLSTAT (v2019.1.3) was used to analyze the data in an ANOVA combined with Tukey’s test for pairwise differences. Significantly different attributes (*p* < 0.05) were selected based on the ANOVA combined with Tukey’s test and analyzed further by Principal Component Analysis (PCA) using PanelCheck V1.4.2 (Norway).

## Results and Discussion

### Stress Experiment

**FIGURE 1:**
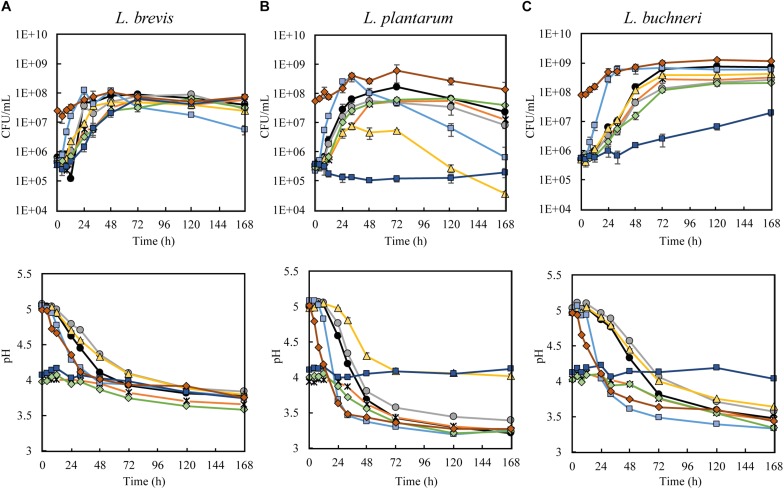
Growth kinetics (upper diagrams) and pH measurements (lower diagrams) during fermentation of wort medium with **(A)** *L. brevis*, **(B)** *L. plantarum*, and **(C)** *L. buchneri* during the reference trial (black line), ethanol trial (5% ethanol, gray), low pH (initial pH 4) obtained with lactic acid trial (orange line) or HCl (green line), iso-α acids trial (5 mg/L, yellow line), high temperature trial (30°C, light blue line), multi-stress trial (5% ethanol, initial pH 4 by lactic acid, 5 mg/L iso-α acids, dark blue line) and high inoculation trial (10^8^ cells/mL, red line).

Initial screenings with *L. brevis L. plantarum*, and *L. buchneri* were conducted to evaluate the effect of different beer-related stress factors on their growth and production of metabolites. The three investigated lactobacilli exhibited good fermentation performance in wort medium at reference conditions (Figures 1A–C), with an increase of 10^2^ CFU/mL within the first 48 h. At the final sampling (after 7 days of fermentation), the observed CFU/mL was 3.9 × 10^7^ for *L. brevis*, 2.4 × 10^7^ for *L. plantarum*, and 7.0 × 10^8^ for *L. buchneri*. These findings are in agreement with previous studies reporting that the nutrient sources present in malt-based media are favorable to lactobacilli growth (Charalampopoulos et al., 2002). A concurring pH drop was observed during fermentation at reference conditions for all LAB strains (Figures 1A–C). The largest pH reduction was obtained with *L. plantarum*, where a final pH of 3.2 was obtained after 7 days in the reference trial. *Lactobacillus buchneri* reached pH 3.5 at the corresponding conditions, while *L. brevis* reached pH 3.7. Elevated growth rate and faster pH reduction was observed for all strains at higher temperature, but higher temperature had no effect on the final pH. This agrees with previous literature, where faster pH-drop, but equal final pH was associated with LAB fermentations at higher temperatures (Narvhus et al., 1998; Østlie et al., 2005).

Generally, the performance of *L. brevis* appeared to be more robust toward different stressor conditions, compared to the two other lactobacilli. The growth of *L. brevis* (Figure 1A) appeared unaffected by iso-α acids alone, or in combination with ethanol and reduced pH (lactic acid) in the multi-stressor condition. The growth of *L. plantarum* (Figure 1B) was severely affected by the presence of iso-α acids and no growth was observed under the multi-stressor condition. *L. buchneri* growth (Figure 1C) was seemingly unaffected by iso-α acids alone, but severely hampered in the multi-stressor condition. The same trend was seen in pH development during fermentation, where a final pH of 3.7-3.8 was reached by *L. brevis* at all stressor conditions (Figure 1A). For *L. plantarum* the pH remained unchanged during fermentation in the multi-stressor trial as it did not grow in these conditions, while a reduction from pH 5 to pH 4 was observed in the iso-α acid trial (Figure 1B, lower). *L. buchneri* was able to generate only a very slight reduction in pH in the multi-stressor trial, from pH 4.1 to pH 4.0, and a reduction from 5 to 3.6 in the presence of iso-α acids alone (Figure 1C).

Based on the growth performance and the ability to reduce pH, the presence of iso-α acids in wort appeared as the most stressful of the investigated environmental factors, especially when coinciding with other brewing related stress-factors, such as low pH and presence of ethanol. The antimicrobial action by iso-α acids, is due to their properties as ionophores that dissipate the transmembrane proton gradient in cells, and by this disrupt the proton motive force and impair cell metabolism (Simpson and Smith, 1992; Simpson, 1993a, b; Ye et al., 1994). Iso-α acids affect microbial cells synergistically with low pH (Simpson and Hammond, 1991; Suzuki, 2011), and increased inhibition in the multi-stressor trial was thus expected. The ability to sustain in an environment with iso-α acids has been associated with the genes *hitA* (Hayashi et al., 2001), *horA* (Sami et al., 1997), and *horC* (Suzuki et al., 2005). The greater resistance toward iso-α acids by *L. brevis* was in accordance with expectations, as *hitA*, *horA* and *horC* have all been identified in *L. brevis* BSO 464. Overall, the higher robustness displayed by *L. brevis* was expected, as the ability of this strain to grow in beer has been demonstrated previously (Bergsveinson et al., 2015, 2016).

### ASCA Analyses of Metabolites Produced During Fermentation in Brewing-Related Stresses

**FIGURE 2:**
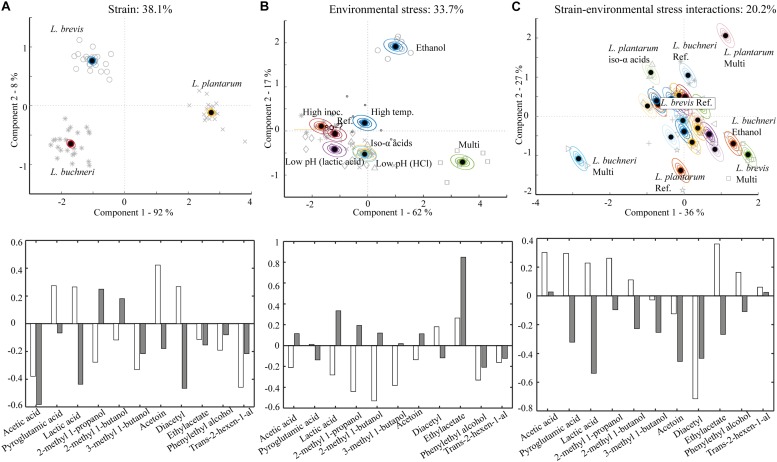
Metabolite variation in samples and replicate variation described by ASCA scores. The model is based on the metabolic composition at the end of fermentation (day 7) with the different lactobacilli strains grown under varying stressor conditions. **(A)** Strain related variation, accounting for 38.1% of the variation in the metabolic data displayed in a score plot (upper) with corresponding loadings (lower). White bars show loadings for component 1 (92%) and gray bars show loadings for component 2 (8%). **(B)** Environmental stressor related variation in the metabolic data, accounting for 33.7% of the variation visualized in a score plot (upper) with corresponding loadings (lower). White bars show loadings for component 1 (62%) and gray bars show loadings for component 2 (17%). **(C)** Strain-environmental stress interaction related variation, accounting for 20.2% of the metabolic variation, visualized in a score plot (upper) with corresponding loadings (lower). White bars show loadings for component 1 (36%) and gray bars show loadings for component 2 (27%).

Metabolites generated during fermentation by the different lactobacilli in wort with different stressor conditions were analyzed and visualized in ASCA plots (Figure 2). The factor “strain” accounted for 38% of the variation in the metabolic data and was the most important variable (Figure 2A). This is consistent with previous reports showing that production of metabolites by lactobacilli is strain-dependent (Cui et al., 2019). A clear separation of the metabolic profile of the three lactobacilli strains is observable in the plot (Figure 2A); component 1 explained 92% of the strain variation in the data-set, while component 2 explained the remaining 8%. The loading weights (Figure 2A) showed that metabolites driving the strain-related variation both in component 1 and 2 included lactic acid, acetic acid and diacetyl. The production of these metabolites was significantly different between all three lactobacilli strains (Supplementary Figures S1A–C). *L. plantarum* generated the highest amount of lactic acid (4181 mg/L) and diacetyl (1.1 mg/L), but the lowest amount of acetic acid (208 mg/L) in the reference trial. At the same conditions *L. brevis* produced the lowest amount of lactic acid (1195 mg/L), no diacetyl and 382 mg/L acetic acid. *L. buchneri* did not produce diacetyl but generated 1822 mg/L lactic acid and the highest quantity of acetic acid (703 mg/L) in the reference trial. Higher relative production of acetic acid by *L. brevis* and *L. buchneri* was expected as these are both obligately heterofermentative, while *L. plantarum* is facultative heterofermentative and produces primarily lactic acid from hexose catabolism (Von Wright and Axelsson, 2019).

The factor “environmental stress” explained 33.7% of the variation in the metabolic dataset (Figure 2B). All stressor conditions, except high inoculation, yielded metabolic compositions significantly different from the reference trial, as shown by separation of the various conditions in the ASCA score plot. The multi-stressor and ethanol trial were the most influential stresses with respect to metabolic composition. The most important driver of component 1 (62% variation in the model) was 2-methyl 1-butanol and the most important driver of component 2 (17% of the variation in the model) was ethylacetate (Figure 2B). The production of 2-methyl 1-butanol was reduced in the multi-stressor trial for all three lactobacilli compared to the reference trial (Supplementary Figure S1I). This might result from a slower metabolism for *L. plantarum* and *L. buchneri*, as the growth of both these was severely affected in the multi-stressor trial (Figures 1B,C). Even though no pronounced effect on the growth of *L. brevis* was observed (Figure 1A), the metabolic activity could still be influenced as observed for the production of 2-methyl 1-butanol. The concentration of ethylacetate increased in the ethanol trial for all lactobacilli compared to the reference trial (Supplementary Figure S1D). Ethanol is a substrate for enzymatic ethylacetate synthesis (Kallel-Mhiri and Miclo, 1993; Costello et al., 2013) thus an increased ethanol concentration in beer is expected to facilitate synthesis of ethylacetate (Cristiani and Monnet, 2001; Liu et al., 2004).

The factor “interactions between strain and environmental stress” accounted for 20% of the variation in the metabolic data (Figure 2C). The multi-stressor condition was most influential with respect to metabolite production for the three lactobacilli; the second most influential stressor was iso-α acids for *L. plantarum* and ethanol for *L. buchneri*. Lactic acid, diacetyl and ethylacetate were all important contributors to component 1 and 2 in the strain-stressor interaction related ASCA (Figure 2C). While none of the stressors yielded significant changes in lactic acid production for *L. brevis*, all stressor conditions resulted in higher or lower quantities of lactic acid produced by *L. plantarum* (Supplementary Figure S1A) compared to the reference trial. Higher lactic acid production was obtained in the higher inoculation level or higher temperature trial for *L. buchneri*, while the multi-stressor condition resulted in a significant reduction of lactic acid production for *L. buchneri* compared to the reference trial (Supplementary Figure S1A). Lactic acid is the main metabolite generated by lactobacilli (Ibrahim and Ouwehand, 2019), and the strain-dependent difference in shifts in lactic acid yield mirrors the growth and pH-development results. *L. brevis* did not produce diacetyl under any conditions, while *L. plantarum* did in all trials (Supplementary Figure S1C). Fermentation with *L. plantarum* at lower pH (HCl), in the multi-stressor trial, higher inoculation rate and high incubation temperature led to a decrease of diacetyl in comparison with the fermentation at reference conditions from 1.1 ± 0.1 mg/L to 0.3–0.9 ± 0.1 mg/L. The highest concentration of diacetyl across all strains and stressor conditions was generated by *L. buchneri* in the multi-stressor trial, yielding a final concentration of 2.1 ± 0.1 mg/L. Diacetyl is associated with caramel and buttery flavors (Harrison, 1970). It is generally regarded as an off-flavor in beer where the reported detection limit is as low as 0.1 mg/L (Vann and Sheppard, 2005). The diacetyl produced by *L. buchneri* was well above the reported sensory threshold and would likely be sensorially influential if the fermented wort medium were to be tasted. Ethylacetate production was stimulated by ethanol for all strains (Supplementary Figure S1D). Ethylacetate is associated with fruity and solvent-like flavors (Meilgaard et al., 1979) and the reported sensory threshold in beer is 30 mg/L (Harrison, 1970). The generated quantities, of less than 1 mg/L for all lactobacilli (Supplementary Figure S1), were therefore below sensory thresholds.

The results from the stressor trials demonstrated that all tested strains could produce metabolites relevant for sour beer production when fermenting wort medium. Differences in tolerance toward beer related stress resulted in different metabolic profiles. *L. brevis* was the most stress resistant strain, while *L. plantarum* displayed the lowest stress tolerance. All lactobacilli generated substantial quantities of organic acids and reduced the pH of wort medium and are thus candidates for sour beer production. However, the data suggest that attention should be paid to strain selection in conjunction with production method. A clear example of this is the high production of diacetyl by *L. buchneri* in the multi-stress trial. Diacetyl is generally considered an off-flavor in beer. The high production in presence of multiple stressors, could mean that *L. buchneri* is unsuited for sour beer fermentation in presence of multiple stresses. The applied *L. buchneri* strain could, however, be suited for a kettle sour approach, where LAB grows in unhopped wort without beer stress such as ethanol, iso-α acids and low pH (Tonsmeire, 2014).

### Small Scale Co-fermentation Experiment

**FIGURE 3:**
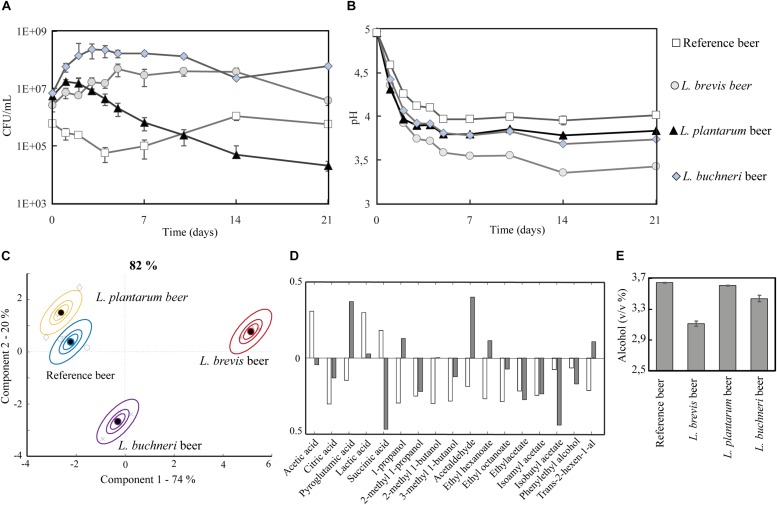
Small scale fermentations (400 mL) of reference, *L. brevis*, *L. plantarum*, and *L. buchneri* beers during 21 days of incubation at 22°C. **(A)** Growth of *S. cerevisiae* in the reference beer, *L. brevis* in the *L. brevis* beer, *L. plantarum* in the *L. plantarum* beer and *L. buchneri* in the *L. buchneri* beer. Note that while S. cerevisiae was present in all four beers but only the growth curve in the reference beer is displayed. The *S. cerevisiae* growth curve was similar for yeast alone and in co-fermentation with LAB, and all four *S. cerevisiae* growth curves are displayed in Supplementary Figure S2. **(B)** pH development. **(C)** Metabolite variation in samples and replicate variation described by ASCA scores. The model is based on the metabolite composition at the end of fermentation (day 21). The model explains 82.4% of the variation in metabolites. **(D)** Loading for ASCA model in panel **(C)**. White bars show loadings for component 1 (74.3%) and gray bars show loadings for component 2 (19.8%). **(E)** Final alcohol percentage (day 21).

A lab-scale experiment (400 mL) where the three different lactobacilli were inoculated simultaneously with *S. cerevisiae* was conducted to investigate how the bacteria performed during co-fermentation for 3 weeks. The co-fermentation product by *S. cerevisiae* with *L. brevis, L. plantarum*, and *L. buchneri* is hereafter referred to as the “*L. brevis* beer,” “*L. plantarum* beer,” and “*L. buchneri* beer.” A reference fermentation with *S. cerevisiae* alone, referred to as “the reference beer,” was also carried out. The growth medium was wort of the same composition as in the stressor experiments, supplemented with 5 mg/L iso-α acids. The presence of *S. cerevisiae* did not affect lactobacilli viability as their cell counts were similar between single cultures (in the presence of iso-α acids) and co-cultures with *S. cerevisiae* (Figure 3A). Vice versa, the acidic environment imposed by the lactobacilli was not detrimental to the yeast viability and no pronounced effect was observed between reference single strain beer culture and mixed strain culture beers (Supplementary Figure S2). For all four fermentations, a primary pH drop occurred within the five first days (Figure 3B); the final pH was lower in all co-fermentations with lactobacilli compared to the reference beer. The final pH in the reference beer was 4.0, compared to pH 3.4 for *L. brevis* beer, 3.8 for *L. plantarum* beer and 3.7 for *L. buchneri* beer. All lactobacilli were thus able to generate sour beers in the employed co-fermentation method, according to the definition suggested by Tonsmeire (2014) of beer with pH 3.1–3.9. *L. brevis* emerged as the more resistant strain with respect to beer fermentation, as it generated the lowest pH in co-fermentation with *S. cerevisiae*.

All lactobacilli influenced the final metabolite composition after co-fermentation. As observed for pH development, *L. brevis* appeared as the most influential with regards to metabolite composition (Figure 3C and Supplementary Table S2). The ASCA model explained 82% of the variation in the metabolites, where the *L. brevis* beer was separated from the others in component 1 (74.3% of the variation in the model) and the *L. buchneri* beer was separated from the others in component 2 (18.9% of the variation in the model). Lactic and acetic acid were the most important drivers of component 1, while succinic acid and isoamyl acetate were important drivers of component 2 (Figure 3D). The lowest quantities of organic acids were obtained in the reference beer (111 ± 12 mg/L acetic, and lactic and succinic acid below detection, Supplementary Table S2) followed by the *L. plantarum* beer (124 ± 3 mg/L acetic, 531 ± 26 mg/L lactic and succinic acid below detection, Supplementary Table S2) and the *L. buchneri* beer (423 ± 17 mg/L acetic, 878 ± 38 mg/L lactic and 167 ± 5 mg/L succinic acid, Supplementary Table S2). The highest quantities of organic acids were obtained in the *L. brevis* beer (951 ± 25 mg/L acetic, 2300 ± 55 mg/L lactic, and 110 ± 3 mg/L succinic acid). The greater influence exerted by *L. brevis* was not only due to its superior stress tolerance and ability to carry out its metabolism during co-fermentation, but also due to an inhibiting effect on *S. cerevisiae* metabolism. This effect was evident from the reduced ethanol production (Figure 3E), reduced apparent degree of fermentation (ADF) (Supplementary Figure S3) and reduced production of a number of metabolites typical for *S cerevisiae* (isoamyl acetate, 2- methyl 1-propanol, 3-methyl 1-butanol, 2-methyl 1-butanol, 1 propanol) in the *L. brevis* beer compared to the three other beers (Supplementary Table S2).

### Beer Production Through Co-fermentation

**FIGURE 4:**
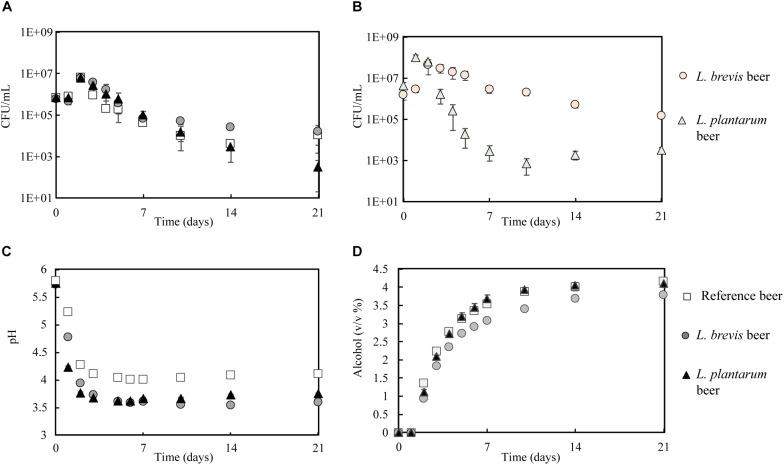
Fermentation (10 L scale) in reference, *L. brevis* and *L. plantarum* beers during 21 days of fermentation at 22°C. **(A)** *S. cerevisiae* growth. **(B)** Lactobacilli growth in *L. brevis* and *L. plantarum* beers. **(C)** pH development. **(D)** Ethanol development.

Based on the results from the stress experiment, where undesirable accumulation of diacetyl was produced by *L. buchneri* in the multi-stressor trial (Supplementary Figure S1C), only *L. brevis* and *L. plantarum* were selected for upscaled beer production. In order to determine how the strains performed at larger scale in a brewery-like setting, 10 L fermentations at 22°C were conducted using wort. The growth patterns of the lactobacilli were comparable to the small-scale co-fermentations (Figure 4B). *S. cerevisiae* growth was consistent with the small-scale co-fermentation in the respect that the CFU/mL were similar regardless of lactobacilli presence (Figure 4A). The final sampling (21 days) was an exception, where the CFU/mL for *S. cerevisiae* were lower in co-fermentation with *L. plantarum* (3.3 × 10^2^ CFU/mL) compared to *S. cerevisiae* alone (1.1 × 10^4^ CFU/mL) or with *L. brevis* (0.7 × 10^4^ CFU/mL).

The pH in all three fermentations dropped, with the majority of the pH reduction occurring during the five first days (Figure 4C). As for the small-scale co-fermentation, *L. brevis* generated the largest pH-drop, with a final pH of 3.6, compared to 3.8 for *L. plantarum* and 4.1 for in the reference beer. Ethanol was produced all throughout the different fermentations (Figure 4D), reaching a final concentration of 4.2% (v/v) in the reference, 4.1% in the *L. plantarum* beer and 3.8% for *L. brevis* beer. As for the small scale-co-fermentation, a correspondingly lower ADF was observed in the *L. brevis* beer (Supplementary Figure S4). It could be argued that the minor reduction in ethanol production by yeast was merely a result of reduced fermentation pH, as the final pH was lower in the *L. brevis* co-fermentation. However, in a study by Alcine Chan et al. (2019) looking at co-fermentation with *L. paracasei* L26 and *S. cerevisiae* S-04, no significant effect from bacterial co-fermentation on ethanol production was found. In a previous study we have tested for an eventual pH-effect by adding lactic acid to wort prior to yeast fermentation. No effect by reduced fermentation pH (final beer pH 3.7) was found on ethanol production or ADF for the same yeast strain (Dysvik et al., 2019). These results suggest that the reduced performance by *S. cerevisiae* with respect to ethanol production and ADF in the *L. brevis* co-fermentation, was caused by some effects exerted by the presence of *L. brevis* itself. The results also demonstrate the importance of the strain to be used in order to obtain wanted properties in the final product. In the current study, *L. brevis* generated higher quantities of organic acids (Supplementary Table S3) and would be the best choice if low pH/high organic acid content in beer was wanted. However, *L. brevis* impaired the yeast metabolism in some way, and thereby generates a beer where fermentable sugars in the wort was exploited to a lower extent.

The amount of amino acids and carbohydrates throughout the fermentations was determined in order to assess the utilization patterns by the different lactobacilli (Table 1). *L. plantarum* contributed to a higher initial depletion of all amino acids compared to the *L. brevis* beer and the reference beer, as the quantities of amino acids were lower after 1 day in the *L. plantarum* beer. An exception was the amount of arginine, which was lower after 1 day in the *L. brevis* beer (20 μmol/g) compared to the *L. plantarum* beer (26 μmol/g) and the reference (43 μmol/g). The concentration of most amino acids was similar after 21 days in the *L. plantarum* beer and the reference beer; The *L. brevis* beer however, contained higher quantities of the amino acids alanine, glycine, histidine, tyrosine and phenylalanine. Both lactobacilli seemingly produced γ-aminobutyric acid (GABA), as this amino acid accumulated in the co-fermented beers (Table 1). The increase was more pronounced in the *L. brevis* beer, where the GABA concentration reached 0.68 μmol/g in 21 days. GABA is produced from glutamic acid (Ueno, 2000) and production by lactobacilli has been proven previously (Choi et al., 2006; Li and Cao, 2010). GABA is a product of the glutamate decarboxylase (Piornos et al., 2019) system (GAD), which represents an acid stress response exerted by lactobacilli (Higuchi et al., 1997). In the GAD system, decarboxylation of glutamic acid increases the intracellular pH by consuming a proton. The arginine deaminase (ADI) pathway can also be part of the response toward acid stress in lactobacilli (Champomier Verges et al., 1999). In the ADI pathway, ATP is generated as arginine is converted to ornithine, carbon dioxide and ammonia. The elevated depletion of arginine, and the production of ornithine (0.29 μmol/g after 21 days) in the *L. brevis* beer, suggests that the ADI pathway is active in *L. brevis* as reported previously in heterofermentative LAB (e.g. *L. brevis*) (Liu et al., 1995). Taken together, these results indicate higher activity of the GAD and ADI systems in *L. brevis* compared to *L. plantarum* and provide an explanation of the increased impact of *L. brevis* during co-fermentation with *S. cerevisiae*. Notably, ingestion of GABA has been associated with a number of health benefits (Dhakal et al., 2012); GABA is therefore classified as a bioactive compound (Chou and Weimer, 1999) and may be a beneficial compound in beer. Amino acid metabolism is essential for flavor formation, both by *S. cerevisiae* (Hernández-Orte et al., 2006) and lactobacilli (Smit et al., 2005), and amino acid availability can be directly correlated with production of compounds such as esters and higher alcohols. The amino acid metabolism is highly complex, even in single strain fermentations, as the same metabolic intermediates are involved multiple metabolic pathways (Fairbairn et al., 2017). Prediction of the sensory output from the amino acid composition of wort, resulting from a mixed fermentation with *S. cerevisiae* and *Lactobacillus* is not possible, and a through discussion of this is beyond the scope of the current study. It should however be noted, that the difference observed in the amino acid degradation patterns for all three beer fermentations, will affect the output of flavor active metabolites. This is in all likelihood part of the explanation for differences in final metabolite composition and sensory properties discussed in the following sections.

**TABLE 1:** Free amino acids and carbohydrates during fermentation (10 L, 22°C, 21 days) of the reference, *L. brevis* and *L. plantarum* beers.

Amino acid	Reference beer			*L. brevis* beer			*L. plantarum* beer		
			
	0 h	1 day	21 days	0 h	1 day	21 days	0 h	1 day	21 days
**Amino acids (μmol/g)**									
*Alanine*	0.85 ± 0.08	0.91 ± 0.09	0.14 ± 0.02	0.90 ± 0.01	0.89 ± 0.03	0.57 ± 0.02	0.88 ± 0.03	0.51 ± 0.03	0.33 ± 0.01
*Glycine*	0.29 ± 0.02	0.29 ± 0.03	0.12 ± 0.01	0.30 ± 0.00	0.30 ± 0.01	0.26 ± 0.01	0.29 ± 0.01	0.17 ± 0.01	0.12 ± 0.01
*Valine*	0.61 ± 0.06	0.63 ± 0.06	0.06 ± 0.01	0.66 ± 0.02	0.65 ± 0.03	0.09 ± 0.01	0.64 ± 0.03	0.37 ± 0.02	0.08 ± 0.01
*Arginine*	0.47 ± 0.05	0.43 ± 0.04	0.04 ± 0.01	0.47 ± 0.00	0.20 ± 0.01	n.d	0.48 ± 0.02	0.26 ± 0.02	0.09 ± 0.01
*GABA*	0.41 ± 0.04	0.42 ± 0.04	0.29 ± 0.01	0.43 ± 0.01	0.48 ± 0.02	0.68 ± 0.04	0.42 ± 0.02	0.25 ± 0.02	0.44 ± 0.01
*Aspartic acid*	0.40 ± 0.04	0.38 ± 0.03	0.04 ± 0.01	0.42 ± 0.00	0.39 ± 0.02	0.09 ± 0.00	0.40 ± 0.01	0.21 ± 0.01	0.06 ± 0.01
*Glutamic acid*	0.52 ± 0.05	0.50 ± 0.04	0.05 ± 0.01	0.55 ± 0.01	0.47 ± 0.02	0.06 ± 0.00	0.54 ± 0.02	0.31 ± 0.02	0.05 ± 0.00
*Asparagine*	0.51 ± 0.06	0.42 ± 0.04	0.02 ± 0.01	0.55 ± 0.01	0.44 ± 0.02	0.02 ± 0.00	0.52 ± 0.02	0.24 ± 0.02	0.03 ± 0.00
*Serine*	0.43 ± 0.04	0.35 ± 0.03	0.05 ± 0.01	0.46 ± 0.00	0.35 ± 0.01	0.05 ± 0.00	0.45 ± 0.02	0.20 ± 0.01	0.04 ± 0.00
*Glutamine*	0.28 ± 0.03	0.24 ± 0.02	0.04 ± 0.00	0.30 ± 0.00	0.21 ± 0.01	0.04 ± 0.00	0.29 ± 0.01	0.13 ± 0.01	0.04 ± 0.00
*Histidine*	0.23 ± 0.03	0.22 ± 0.02	0.06 ± 0.00	0.23 ± 0.00	0.23 ± 0.01	0.11 ± 0.01	0.23 ± 0.01	0.13 ± 0.01	0.08 ± 0.00
*Threonine*	0.27 ± 0.03	0.20 ± 0.02	0.03 ± 0.01	0.28 ± 0.00	0.21 ± 0.01	0.03 ± 0.00	0.27 ± 0.01	0.12 ± 0.01	0.04 ± 0.00
*Citrulline*	n.d.	0.01 ± 0.00	0.01 ± 0.00	0.03 ± 0.00	0.09 ± 0.00	0.05 ± 0.00	n.d	0.01 ± 0.00	0.01 ± 0.00
*Tyrosine*	0.33 ± 0.03	0.33 ± 0.03	0.04 ± 0.01	0.35 ± 0.00	0.33 ± 0.01	0.10 ± 0.01	0.34 ± 0.01	0.19 ± 0.01	0.05 ± 0.00
*Methionine*	0.08 ± 0.01	0.08 ± 0.01	0.04 ± 0.03	0.08 ± 0.00	0.10 ± 0.00	0.08 ± 0.00	0.08 ± 0.00	0.05 ± 0.00	0.04 ± 0.03
*Isoleucine*	0.33 ± 0.03	0.33 ± 0.03	0.04 ± 0.01	0.36 ± 0.01	0.34 ± 0.01	0.03 ± 0.00	0.35 ± 0.01	0.19 ± 0.01	0.05 ± 0.01
*Tryptophane*	0.55 ± 0.06	0.54 ± 0.05	0.04 ± 0.01	0.59 ± 0.01	0.45 ± 0.16	0.05 ± 0.00	0.57 ± 0.02	0.25 ± 0.08	0.07 ± 0.00
*Phenylalanine*	0.21 ± 0.02	0.21 ± 0.02	0.03 ± 0.01	0.23 ± 0.01	0.31 ± 0.13	0.11 ± 0.01	0.22 ± 0.01	0.18 ± 0.09	0.02 ± 0.00
*Leucine*	0.65 ± 0.07	0.57 ± 0.05	0.07 ± 0.01	0.70 ± 0.01	0.60 ± 0.02	0.07 ± 0.00	0.68 ± 0.03	0.34 ± 0.02	0.11 ± 0.01
*Ornithine*	0.14 ± 0.08	0.13 ± 0.08	0.12 ± 0.07	0.20 ± 0.00	0.17 ± 0.01	0.29 ± 0.02	0.20 ± 0.01	0.11 ± 0.00	0.04 ± 0.01
*Lysine*	0.35 ± 0.04	0.27 ± 0.03	0.03 ± 0.00	0.38 ± 0.01	0.27 ± 0.01	0.03 ± 0.00	0.37 ± 0.02	0.15 ± 0.01	0.04 ± 0.01
**Carbohydrates (g/L)**									
*Glucose*	6.97 ± 0.17	4.95 ± 0.15	0.01 ± 0.00	6.83 ± 0.10	5.11 ± 0.24	0.01 ± 0.00	6.98 ± 0.36	3.08 ± 0.09	0.02 ± 0.01
*Fructose*	3.21 ± 0.19	2.82 ± 0.07	0.18 ± 0.01	3.49 ± 0.05	3.15 ± 0.17	0.14 ± 0.01	4.35 ± 0.12	2.13 ± 0.14	0.14 ± 0.01
*Sucrose*	1.16 ± 0.09	n.d.	n.d.	1.40 ± 0.30	n.d.	n.d.	1.19 ± 0.12	n.d.	n.d.
*Maltose*	52.87 ± 0.87	46.40 ± 3.46	1.08 ± 0.10	52.88 ± 0.91	47.84 ± 1.35	3.12 ± 0.07	54.84 ± 1.02	33.14 ± 0.82	1.20 ± 0.02
*Maltotriose*	11.70 ± 0.78	9.36 ± 0.33	0.64 ± 0.03	13.23 ± 0.30	10.56 ± 0.74	2.85 ± 0.13	14.45 ± 0.35	7.97 ± 0.60	0.78 ± 0.05

*The initial sampling (0 h), 1 day and the final sampling (21 days) are included in the table. All values are averages of triplicates. “n.d.”, not detected.*

The presence of both lactobacilli influenced the carbohydrate utilization during beer fermentation (Table 1). *L. plantarum* contributed to a quicker depletion of maltose, glucose, fructose and maltotriose within 1 day (Table 1) compared to the reference and *L. brevis* beer, but with similar final concentrations to the reference beer (Table 1). In the *L. brevis* beer, the uptake was seemingly slower, as the concentrations of all carbohydrates (except sucrose which is completely depleted in all beers after 24 h) after 1 day was higher compared to the *L. plantarum* beer. Maltose and maltotriose were the most abundant carbohydrate detected in the final beer and their concentrations were higher in the *L. brevis* beer compared to the reference beer.

Overall, these data suggest that lactobacilli influenced the beer fermentation in different ways. While *L. plantarum* contributes to the fermentation by depleting amino acids and carbohydrates quickly, its presence did not disrupt the yeast fermentation extensively, as the final ethanol concentration and ADF are similar to the reference beer. *L. brevis* did, however, affect the final ethanol concentration and ADF, likely interfering with the yeast ability to metabolize free amino acids and carbohydrates.

### Metabolite Composition and Sensory Analysis

**FIGURE 5:**
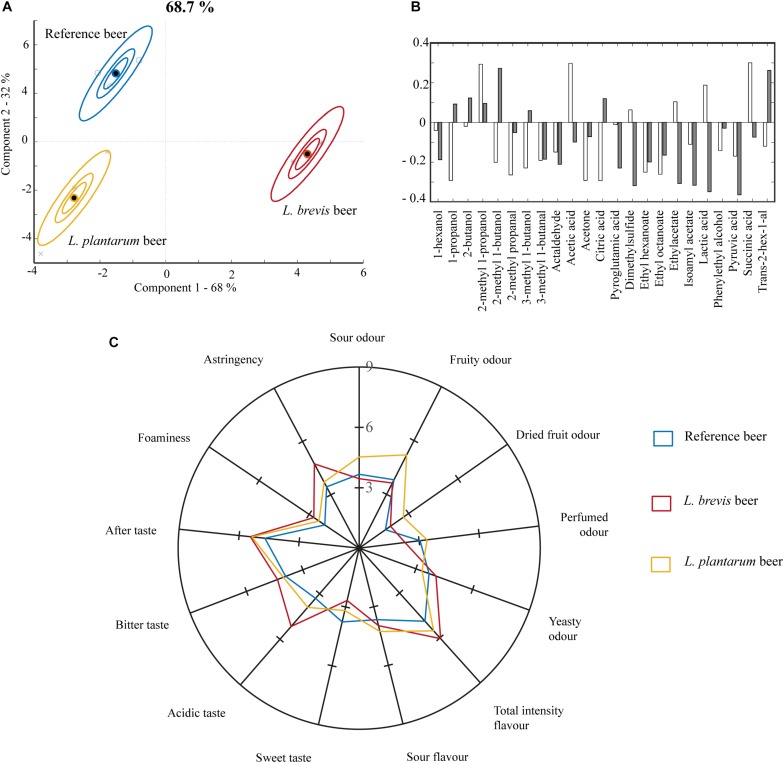
Properties of the final beer from 10 L fermentations at 22°C for 21 days in the reference, *L. brevis* and *L. plantarum* beers. **(A)** Metabolite variation in samples and replicate variation described by ASCA scores. The model is based on the metabolic composition in the final beer products. The model explains 69% of the variation in metabolites. **(B)** Loading weights for ASCA model in panel **(A)**. White bars show loadings for component 1 (68%) and gray bars show loadings for component 2 (32%). **(C)** Sensory properties for the three different beers. The graph only displays scores for sensory attributes assessed as significantly different between two or more of the beers.

The metabolite composition of the final beers was analyzed, and the beers were evaluated sensorially. According to the ASCA score plot on metabolite composition (Figure 5A) and consistently with the small-scale co-fermentation, *L. brevis* exerted more influence in co-fermentation with *S. cerevisiae*, compared to *L. plantarum.* Together component 1 and 2 accounted for 68 and 32% of the variation in the ASCA, respectively, explaining 68.7% of the variation in the metabolites. Lactic acid, acetic acid, ethyl hexanoate and ethyl octanoate were important drivers of the ASCA model, important in both component 1 and 2 (Figure 5B). The highest concentration of organic acids was obtained in the *L. brevis* beer (942 ± 11 mg/L acetic, 2598 ± 56 mg/L lactic, and 196 ± 14 mg/L succinic acid, Supplementary Table S3) followed by the *L. plantarum* beer (89 ± 26 mg/L acetic, 1792 ± 94 mg/L lactic, and succinic acid below detection, Supplementary Table S3), while no lactic acid or succinic acid and only a limited amount of acetic acid (31 ± 4 mg/L, Supplementary Table S3) was generated in the reference beer. Lactic acid is associated with acidity and sourness (Van Oevelen et al., 1976) and has a reported taste threshold of 400 mg/L (Engan, 1974). Acetic acid is associated with acidity, sour (Engan, 1974) and vinegary flavors (Van Oevelen et al., 1976) and has a reported sensory threshold of 200 mg/L (Engan, 1974). Both lactic and acetic acid were well above reported sensory thresholds in the *L. brevis* beer, which corresponds well with this beer being perceived as significantly higher than the *L. plantarum* and the reference beer in acidic taste in the sensory analysis (Figure 5C and Supplementary Table S4). The *L. brevis* beer was scored as significantly higher in astringency compared to the other beers. This corresponds well with the metabolic data, as astringency is partly related to organic acid content (Da Conceicao Neta et al., 2007), and higher perception of astringency is correlated with decreasing pH (Lawless et al., 1996). The *L. plantarum* beer was perceived as highest in sour odor (Figure 5C). The attribute “sour odor” is related to a fresh, balanced odor generally related to presence of organic acids (Supplementary Table S1) (International Organisation for Standardisation [ISO], 2012). The *L. plantarum* and *L. brevis* beers were different in their organic acid content, not only in the total concentrations, but also in the relative ratios between the organic acids in the beer. The lactic:acetic acid ratio in the *L. brevis* beer was approximately 3:1, while the corresponding ratio in the *L. plantarum* beer was closer to 20:1. It could be speculated that this difference in the organic acid content and ratios somehow manifests as a difference in perceived sour odor. The *L. plantarum* beer was scored higher in fruity odor compared to the *L. brevis* and reference beer, and higher in dried fruit odor compared to the reference (Figure 5C and Supplementary Table S4). This corresponds with the beer being higher in the fruity esters ethyl hexanoate and ethyl octanoate (Supplementary Table S3). Ethyl hexanoate is associated with fruit, fennel and solvent flavors (Xu et al., 2017) and has a sensory threshold in beer of 0.3 mg/L (Harrison, 1970). Ethyl octanoate is associated with sweet and fruity flavors (Yonezawa and Fushiki, 2002) and a sensory threshold of 0.9–1.0 mg/L in beer (Pires and Brányik, 2015). At 0.11 ± 0.01 mg/L ethyl hexanoate and 0.03 mg/ethyl octanoate in the *L. plantarum* beer (Supplementary Table S3), both esters were below the sensory threshold. Their presence could, however, be influential to the sensory properties through synergistic, sub-threshold effects (Dalton et al., 2000).

Further significant differences were found between all the three beers in the sensory analysis. Of 23 evaluated attributes (Supplementary Table S1), 13 were scored as significantly different between two or more of the beers (Figure 5C and Supplementary Table S4). Both beers produced through co-fermentation with lactobacilli were perceived as sensorially different from the beer produced through fermentation by *S. cerevisiae* alone. Both the *L. brevis* and *L. plantarum* beers were scored significantly higher in several sensory attributes, compared to the reference beer (Figure 5C). The *L. brevis* and the *L. plantarum* beer were perceived as significantly different from each other in sour odor, fruity odor, perfumed odor, yeasty odor, sweet taste, acidic taste and astringency (Figure 5C and Supplementary Table S4). Note that sour flavor is different from acidic taste, which is one of the basic tastes. Sour flavor is the combined perception of acidic taste together with the retro-nasal contribution from volatile organic acids. Sour flavor is a highly complex sensory property, that is often associated with both freshness and sweet-sour balance.

**FIGURE 6:**
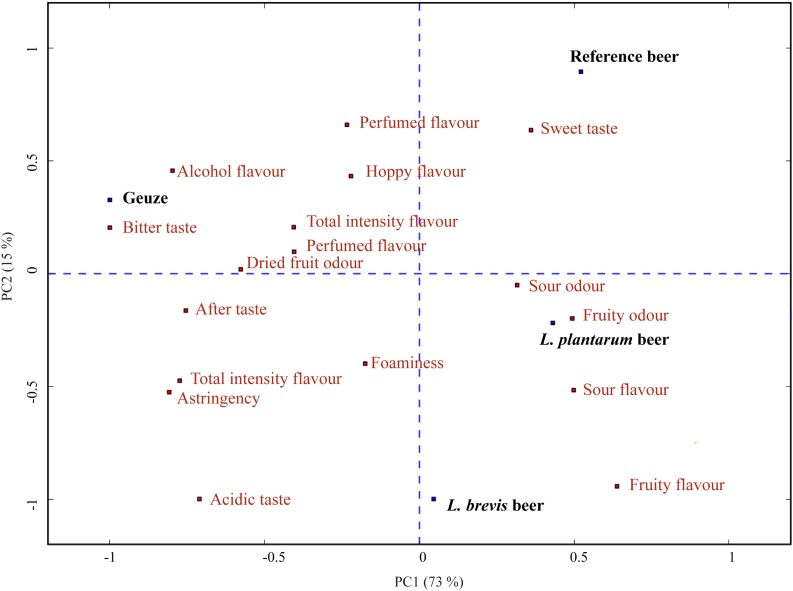
PCA bi-plot with beers as loadings (blue) and attributes as scores (red), based on the sensory analysis of the reference, *L. brevis* and *L. plantarum* beers. The PCA also includes sensory results from a commercial sour beer reference included at the end of the sensory analysis. PC1 explains 73.3% in the sample set, while PC2 explains 15.1%.

In order to compare the sour beers produced in the current study to a traditional sour beer, a commercial Boon Geuze was evaluated at the end of the sensory analysis. To visualize the difference in the flavor profile (Supplementary Table S5) of the four beers, we generated a PCA bi-plot with beers as loadings and sensory attributes as scores (Figure 6). The commercial Geuze beer is oriented oppositely to the three experimentally produced sour beers in component 1 in the PCA plot and was sensorially different. The Geuze was scored as significantly different from all other beers in total odor intensity, alcohol flavor, sour flavor, and fruity flavor (Supplementary Table S5). Being produced through a completely different method, the Geuze was expected to be sensorially different from the beers produced in the current experiment. The decanting technique used for the commercial sour beer prior to sensory evaluation, was applied to avoid the commercial beer being perceived as a completely different product merely due to the difference in carbonation. Geuze beers, such as the commercial sour beer, are typically highly carbonated, while the experimental beers produced were only slightly carbonated. The commercial sour beer was therefore decarbonated to allow assessment of the other sensory attributes without the influence of difference in carbonation level. It should be noted that the introduction of oxygen by the decanting procedure could affect the sensory properties by causing formation of oxidized flavors (Depraetere et al., 2008; Saison et al., 2009). In addition, the decanting might have caused some loss of volatiles and reduced intensity in aroma and odor. However, the sensory results showed that both *L. brevis* and *L. plantarum* sour beers were significantly different from the base beer in multiple sensory attributes and they were both closer than the base beer to the commercial sour beer in attributes such as aftertaste and total flavor intensity. Indeed, the objective of the current study was not to replicate the sensory character of a Geuze style beer, which originates through year-long spontaneous fermentation (Van Oevelen et al., 1977), but rather to get an idea of how beers produced through controlled co-fermentations compared to known commercial sour beers. It is noteworthy that both the *L. brevis* and *L. plantarum* beers were scored closer to the Geuze sour beer, significantly higher compared to the reference beer in total flavor intensity and after taste (Supplementary Table S5). In addition, the *L. brevis* beer was perceived as similar to the Geuze sour beer and significantly different from the *L. plantarum* and reference beer in sweet taste, acidic taste and astringency.

## Conclusion

In conclusion, this study shows that the investigated strains *L. brevis*, *L. plantarum*, and *L. buchneri* displayed different responses to beer-related environmental stress factors. While *L. brevis* was robust toward stress, the metabolism of *L. plantarum* and *L. buchneri* was severely inhibited by multiple environmental stress-factors. The metabolic data revealed how a stressful environment can cause accumulation of unwanted, flavor active metabolic products during fermentation (i.e. accumulation of diacetyl in *L. buchneri* multi-stressor trial). Remarkably, the current study demonstrates how controlled co-fermentation with *S. cerevisiae* and a stress-vulnerable *L. plantarum* can be used to produce sour beer within a 21-day fermentation period, resulting in a product with increased total flavor intensity, fruity odor and dried fruit odor. *L. plantarum* is commercially used in kettle souring where it performs best as no hops or other stress factors that could inhibit its growth are present in the wort. In addition, *L. brevis* was used to produce a sour beer with increased total flavor intensity that was similar to commercial Geuze sour beer in acidic taste, sweet taste and astringency. However, caution should be used in an industrial setting without differential brewing lines as *L. brevis* BSO464 is a common beer-spoilage microorganism. Controlled mixed fermentations, and fermentations of wort with non-traditional microbes, offer a great potential for creation of novel sour beer products with high production control and short production time. By extending the currently explored method to other mixed fermentations with multi-strain yeasts and/or bacteria combinations, it might be possible to shift more sensory properties in the direction of traditional sour beer products and create beer beverages with novel organoleptic properties.

## Data Availability Statement

All datasets generated for this study are included in the article/Supplementary Material.

## Author Contributions

AD, GD, and HØ conceptualized the study. AD, E-OR, BW, and TW were involved in the design of the study and experimental planning. AD conducted the fermentation experiments. KM carried out the sensory analysis. KL performed the statistical analysis. AD wrote the first draft of the manuscript, with contribution of SL. AD, SL, and BW finalized the manuscript and prepared for publication. All authors contributed to manuscript revision and approved the submitted version.

## Conflict of Interest

The authors declare that the research was conducted in the absence of any commercial or financial relationships that could be construed as a potential conflict of interest.
