# Supplementary material for: Co-fermentation Involving Saccharomyces cerevisiae and Lactobacillus Species Tolerant to Brewing-Related Stress Factors for Controlled and Rapid Production of Sour Beer
Source: Front Microbiol. 2020 Feb 21;11:279. doi: 10.3389/fmicb.2020.00279 (PMC7048013; doi:10.3389/fmicb.2020.00279)
Supplement: Supplementary file 1 [file Data_Sheet_1.docx]

**Supplementary Material for**

**Co-fermentation involving *Saccharomyces cerevisiae* and *Lactobacillus* species tolerant to brewing-related stress factors for controlled and rapid production of sour beer**

Anna Dysvik, Sabina Leanti La Rosa, Kristian Hovde Liland, Kristine S. Myhrer, Hilde Marit Østlie, Gert De Rouck, Elling-Olav Rukke, Bjørge Westereng and Trude Wicklund


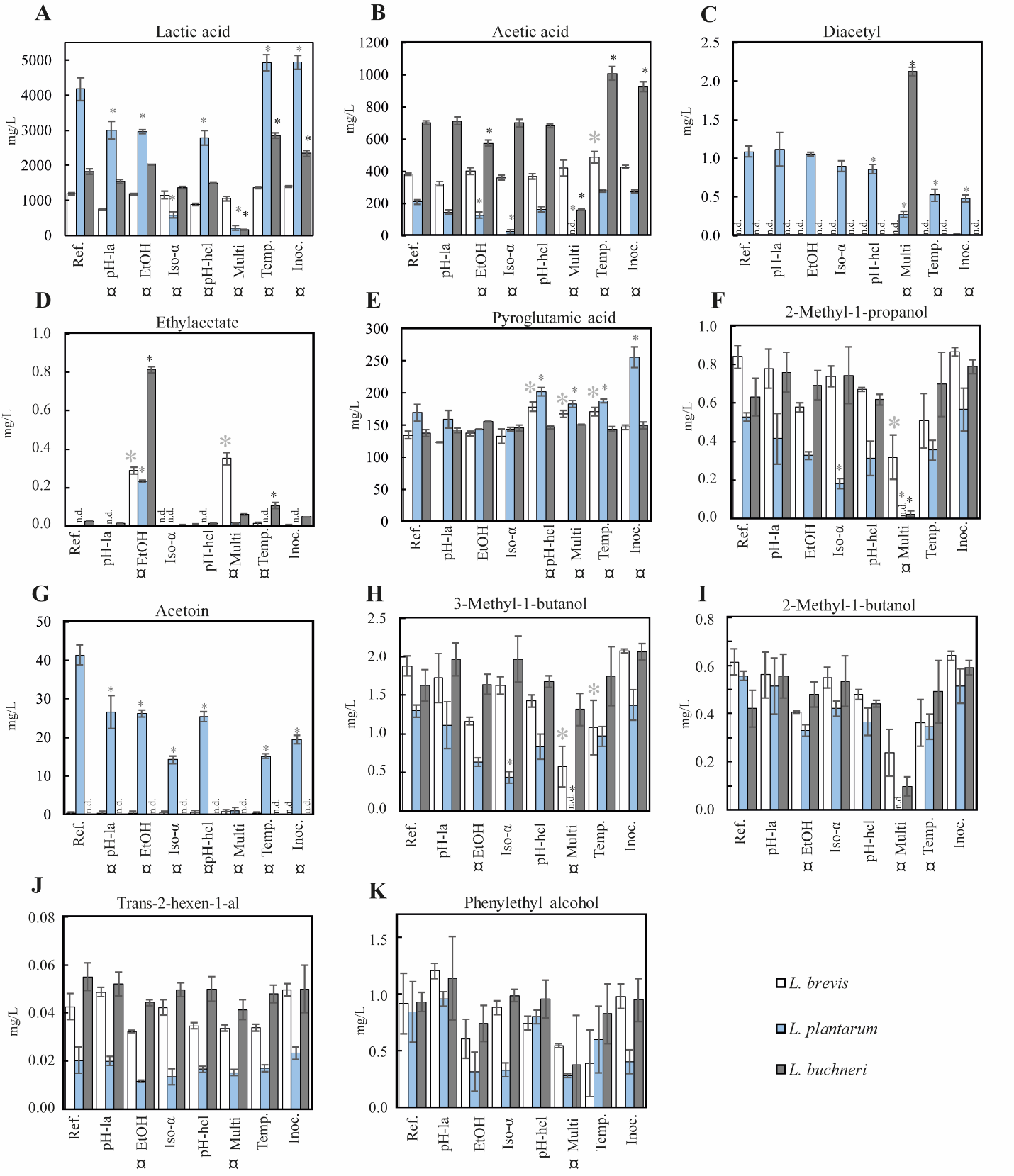


**Figure S1.** Metabolic compounds produced by different lactobacilli after 7 days of fermentation with varying brewing-related stresses. Average concentrations with standard deviation as error bars are presented for **(A)** lactic acid, **(B)** acetic acid, **(C)** diacetyl, **(D)** ethylacetate, **(E)** pyroglutamic acid, **(F)** 2-methyl 1-propanol, **(G)** acetoin, **(H)** 3-methyl 1-butanol, **(I)** 2-methyl 1-butanol, **(J)** trans-2-hexen-1-al and **(K)** phenylethyl alcohol. Ref = reference trial; pH-la, = low initial pH (Lactic acid) trial; EtOH = ethanol trial; iso-α = iso alpha-acid trial; pH-HCl = low initial pH (HCl) trial; Multi = multi stressor trial with ethanol, iso-α acids and low initial pH (lactic acid); Temp. = high temperature trial; Inoc = high inoculum trial. Stressor dependent concentrations across all lactobacilli strains, significantly different to that obtained at the reference condition (significant stressor effect according to ANOVA at significance level p < 0.05) are indicated with ¤ at the stressor. Concentrations significantly different from that obtained by respective strains (significant strain-stressor interactions according to ANOVA at significance level p < 0.05) are indicated by with * for *L. brevis*, * for *L. plantarum* and * for *L. buchneri*. *n.d* = non detected values.


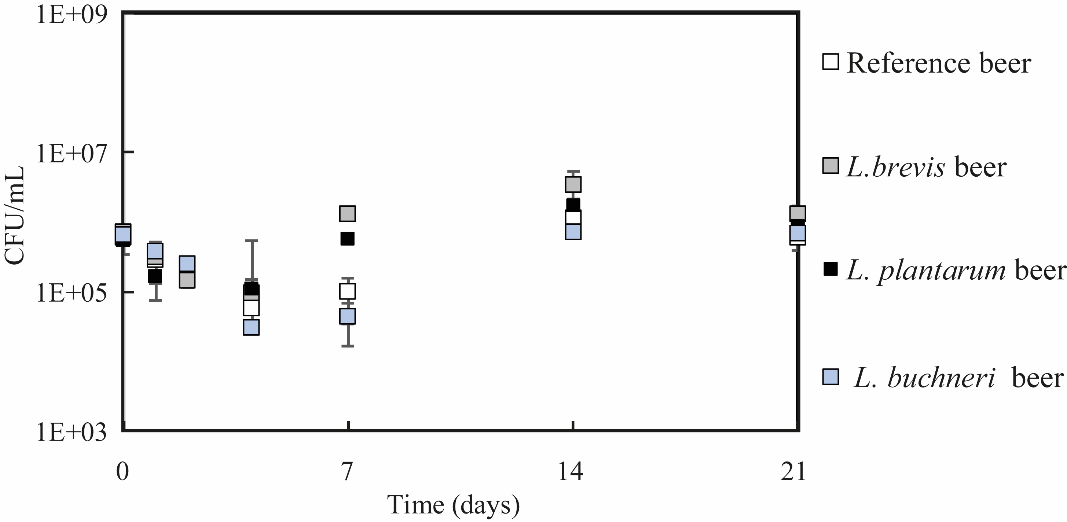


**Figure S2.** Growth of *S. cerevisiae* during 400 mL fermentation (21 days, 22°C) in reference (white squares), *L. brevis* (grey squares), *L. plantarum* (black squares) and *L. buchneri* beers (light blue squares).


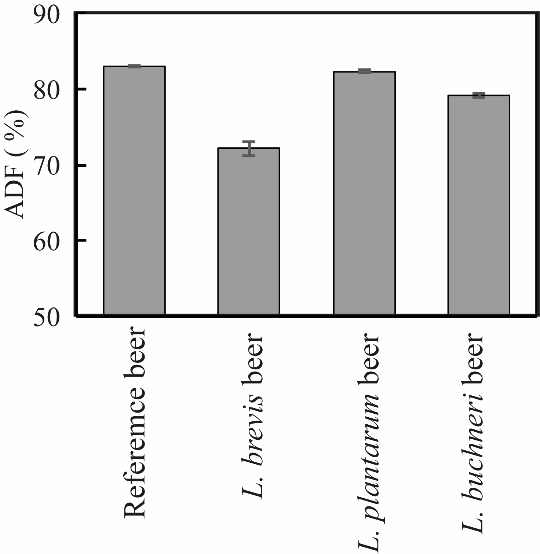


**Figure S3.** Apparent degree of fermentation (ADF) during 400 mL fermentations (22°C, 21 days) in reference, *L. brevis*, *L. plantarum* and *L. buchneri* beers.


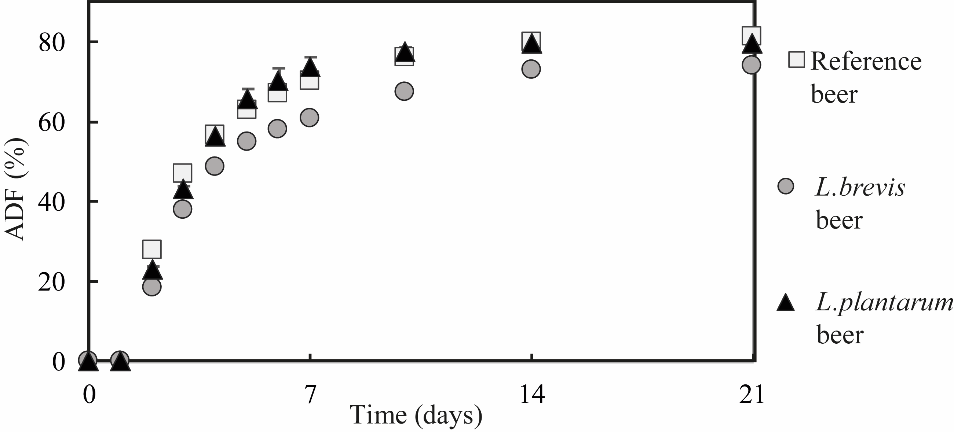


**Figure S4.** Development of apparent degree of fermentation (ADF) during 10 L fermentations (22 °C, 21 days) in reference (white squares), *L. brevis* (grey circles) and *L. plantarum* (black triangles) beers*.*

**Table S1.** Attributes (odour (O), texture, taste (T), and flavor (F)) and descriptions used in descriptive sensory analysis of the beers.

| Attribute | Description |  | Attribute | Description |
| --- | --- | --- | --- | --- |
| *Odour* | |  | ***Taste and flavor*** | |
| *Total intensity-O* | The strength of all odours in the sample |  | *Total intensity-F* | The strength of all flavors in the sample |
| *Sour-O* | Related to a fresh, balanced odour due to the presence of organic acids |  | *Sour-F* | Related to a fresh, balanced flavor due to the presence of organic acids |
| *Hoppy-O* | Odour of hops |  | *Sweet-T* | Related to the basic taste sweet (sucrose) |
| *Malty-O* | Odour of malt |  | *Acidic-T* | Related to the basic taste acidic (citric acid) |
| *Fruity-O* | Odour of fruits (citrus, pineapple, pears, apple and rhubarb) |  | *Bitter-T* | Related to the basic taste bitter (caffeine) |
| *Perfume-O* | Odour of flowers and perfume |  | *Hoppy-F* | Flavor of hops |
| *Yeasty-O* | Odour of yeast |  | *Malty-F* | Flavor of malt |
| *Dried fruit-O* | Odour of dried fruits (prunes, apricots, peaches) |  | *Fruity-F* | Flavor of fruits (citrus, pineapple, pears, apple and rhubarb) |
| *Texture* | |  | *Perfume-F* | Flavor of flowers and perfume |
| *Fullness* | Mechanical textural attribute relating to resistance to flow |  | *Yeast-F* | Flavor of yeast |
| *Foaminess* | Mechanical textural attribute related to a foaming, sparkling sensation in the mouth |  | *Alcohol-F* | Flavor of alcohol, spirits (ethanol) |
| *Astringency* | Organoleptic attribute of pure substances or mixtures which produces the astringent sensation |  | *After-F* | Flavor which occurs 30 seconds after elimination of the product |

| Metabolites, *IUPAC names* (mg/L) | Reference |  | *L. brevis beer* |  | *L. plantarum* beer |  | *L. buchneri* beer |  |
| --- | --- | --- | --- | --- | --- | --- | --- | --- |
| Acetic acid, *Acetic acid* | 110.58 ± 11.62 | c | 951.41 ± 24.90 | a | 124.19 ± 2.64 | c | 422.59 ± 17.25 | b |
| Citric acid, *2-hydroxypropane -1,2,3-tricarboxylic acid* | 130.83 ± 0.74 | a | n.d. | c | 126.05 ± 2.41 | a | 118.19 ± 4.19 | b |
| Pyroglutamic acid, *(2S)-5-oxop-yrrolidine-2-carboxylic acid* | 194.09 ± 2.01 | b | 193.11 ± 1.73 | b | 238.42 ± 5.53 | a | 188.67 ± 1.22 | b |
| Lactic acid,  *2-hydroxypropanoic acid* | n.d. | d | 2299.64 ± 55.08 | a | 531.00 ± 26.19 | c | 877.66 ± 38.3 | b |
| Succinic acid, *butanedioic acid* | n.d. | b | 111.76 ± 3.04 | b | n.d. | c | 166.50 ± 4.77 | a |
| 1-propanol, *propan-1-ol* | 14.07 ± 0.70 | a | 7.30 ± 0.56 | c | 14.37 ± 0.67 | a | 10.77 ± 0.42 | b |
| 2-methyl 1-propanol*,*  *2-methylpropan-1-ol* | 19.32 ± 0.65 | a | 16.26 ± 0.60 | b | 19.95 ± 1.07 | a | 20.32 ± 0.50 | a |
| 2-methyl 1-butanol,  *2-methylbutan-1-ol* | 9.97 ± 0.30 | a | 7.70 ± 0.30 | b | 10.36 ± 0.24 | a | 9.52 ± 0.20 | a |
| 3-methyl 1-butanol,  *3-methylbutan-1-ol* | 42.22 ± 1.21 | a | 35.20 ± 1.21 | b | 43.22 ± 1.11 | a | 42.40 ± 0.97 | a |
| Acetaldehyde*,*  *Acetaldehyde* | 24.74 ± 7.35 | ab | 8.50 ± 1.59 | bc | 32.13 ± 8.34 | a | 3.65 ± 0.26 | c |
| Ethyl hexanoate,  *Ethyl hexanoate* | 0.05 ± 0.00 | a | 0.02 ± 0.00 | b | 0.05 ± 0.01 | a | 0.04 ± 0.00 | ab |
| Ethyl octanoate,  *Ethyl octanoate* | 0.03 ± 0.00 | a | 0.01 ± 0.00 | b | 0.03 ± 0.00 | a | 0.03 ± 0.00 | a |
| Ethyl acetate*,*  *Ethyl acetate* | 8.65 ± 0.24 | a | 6.15 ± 0.15 | b | 7.78 ± 1.16 | ab | 8.76 ± 0.45 | a |
| Isoamyl acetate,  *3-methylbutyl acetate* | 0.32 ± 0.02 | a | 0.16 ± 0.01 | b | 0.29 ± 0.06 | a | 0.33 ± 0.03 | a |
| Isobutyl acetate*,  *2-methylpropyl acetate* | 0.01 ± 0.00 |  | 0.01 ± 0.00 |  | 0.01 ± 0.00 |  | 0.02 ± 0.00 |  |
| Phenylethyl alcohol*,  *2-phenylethanol* | 2.11 ± 1.03 |  | 2.16 ± 0.20 |  | 2.81 ± 0.48 |  | 3.00 ± 0.23 |  |
| Trans-2-hexen-1-al*,  *(E)-hex-2-enal* | 0.03 ± 0.00 |  | 0.03 ± 0.00 |  | 0.03 ± 0.00 |  | 0.03 ± 0.00 |  |

**Table S2.** Metabolites after 400 mL fermentations (22 °C, 21 days) in reference, *L. brevis*, *L. plantarum* and *L. buchneri* beers. n.d. = non detected. Significant different concentrations according to ANOVA at p < 0.05 are indicated with different letters, where “a” gives the highest concentration group, “b” the second highest and “c” the lowest concentration group. * p > 0.05

**Table S3.** Metabolites after 10 L fermentations (22 °C, 21 days) in reference, *L. brevis* and *L. plantarum* beers. n.d. = non detected. Significant different concentrations according to ANOVA at p < 0.05 are indicated with different letters, where “a” gives the highest concentration group, “b” the second highest and “c” the lowest concentration group. *p > 0.05.

| Metabolites, *IUPAC names* (mg/L) | *S. cerevisiae* |  | *L. brevis* beer |  | *L. plantarum* beer |  |
| --- | --- | --- | --- | --- | --- | --- |
| Acetic acid, *Acetic acid* | 31.37 ± 3.81 | c | 942.06 ± 10.77 | a | 88.86 ± 25.61 | b |
| Citric acid, *2-hydroxypropane -1,2,3-tricarboxylic acid* | 199.25 ± 4.18 | a | n.d. | c | 176.17 ± 9.93 | b |
| Pyroglutamic acid*, *(2S)-5-oxop-yrrolidine-2-carboxylic acid* | 91.19 ± 3.47 |  | 95.80 ± 2.24 |  | 99.03 ± 8.19 |  |
| Pyruvic acid, *2-oxopropanoic acid* | 9.02 ± 0.83 | b | 19.22 ± 0.80 | b | 105.45 ± 9.13 | a |
| Lactic acid,  *2-hydroxypropanoic acid* | n.d. | c | 2598.02 ± 55.96 | a | 1791.61 ± 94.23 | b |
| Succinic acid, *butanedioic acid* | n.d. | c | 196.38 ± 13.95 | a | n.d. | b |
| 1-propanol, *propan-1-ol* | 15.88 ± 0.62 | a | 10.11 ± 0.28 | b | 15.58 ± 0.71 | a |
| 2-methyl 1-propanol*,*  *2-methylpropan-1-ol* | 21.66 ± 0.56 | b | 25.87 ± 0.49 | a | 19.06 ± 0.46 | c |
| 2-methyl-propanal, *2-methyl-propanal* | 0.01 ± 0.00 | ab | n.d. | b | 0.01 ± 0.0 | a |
| 2-butanol*, *butan-2-ol* | 0.02 ± 0.01 |  | 0.01 ± 0.01 |  | 0.01 ± 0.01 |  |
| 2-methyl 1-butanol,  *2-methylbutan-1-ol* | 8.71 ± 0.36 | a | 7.58 ± 0.13 | b | 8.09 ± 0.06 | ab |
| 3-methyl 1-butanol*,  *3-methylbutan-1-ol* | 28.08 ± 1.02 |  | 25.77 ± 0.40 |  | 28.06 ± 1.16 |  |
| 3-methyl-butanal**, 3-methylbutanal* | 0.01 ± 0.01 |  | 0.01 ± 0.01 |  | 0.01 ± 0.01 |  |
| Acetaldehyde**,*  *Acetaldehyde* | 10.23 ± 3.69 |  | 8.91 ± 4.34 |  | 20.18 ± 7.45 |  |
| 1-hexanol*, *hexan-1-ol* | 0.02 ± 0.00 |  | 0.03 ± 0.01 |  | 0.03 ± 0.01 |  |
| Ethyl hexanoate,  *Ethyl hexanoate* | 0.07 ± 0.01 | b | 0.05 ± 0.00 | b | 0.11 ± 0.01 | a |
| Ethyl octanoate,  *Ethyl octanoate* | 0.02 ± 00.0 | b | 0.01 ± 0.00 | b | 0.03 ± 0.0 | a |
| Ethyl acetate**,*  *Ethyl acetate* | 4.57 ± 0.40 |  | 6.87 ± 0.59 |  | 6.61 ± 1.28 |  |
| Isoamyl acetate,  *3-methylbutyl acetate* | 0.11 ± 0.01 | b | 0.14 ± 0.02 | ab | 0.21 ± 0.05 | a |
| Phenylethyl alcohol*,  *2-phenylethanol* | 2.08 ± 0.31 |  | 1.85 ± 0.21 |  | 2.19 ± 0.28 |  |
| Trans-2-hexen-1-al*,  *(E)-hex-2-enal* | 0.01 ± 0.00 |  | n.d. |  | n.d. |  |
| Acetone*, propan-2-one* | 0.08 ± 0.00 | b | 0.01 ± 0.01 | c | 0.11 ± 0.02 | a |
| Dimethylsulfide*, *methylsulfanylmethane* | n.d. |  | 0.01 ± 0.0 |  | 0.01±0.00 |  |

**Table S4.** Average sensory scores for all attributes evaluated in the descriptive analysis of the reference, *L. brevis* and *L. plantarum* beers. Beers receiving significantly different scores (p-value < 0.05, according to ANOVA analysis) are indicated with different letters, where group “a” has the highest score, group “b” second highest and “c” has the lowest score.

| Attributes | *L. plantarum* beer | *L. brevis* beer | *S. cerevisiae* | p-value | Significant |
| --- | --- | --- | --- | --- | --- |
| Total intensity odour | 5.44 a | 5.30 a | 5.30 a | 0.897 | No |
| Sour odour | 4.53 a | 3.45 b | 3.67 b | 0.007 | Yes |
| Hoppy odour | 3.76 a | 4.11 a | 4.08 a | 0.574 | No |
| Malty odour | 3.59 a | 3.37 a | 3.40 a | 0.699 | No |
| Fruity odour | 5.20 a | 3.63 b | 3.81 b | 0.002 | Yes |
| Dried fruit odour | 2.68 a | 1.90 ab | 1.59 b | 0.008 | Yes |
| Perfumed odour | 3.40 a | 2.29 b | 3.05 a | 0.005 | Yes |
| Yeasty odour | 3.33 b | 4.06 a | 3.69 ab | 0.027 | Yes |
| Total intensity flavor | 5.53 ab | 6.05 a | 4.89 b | 0.001 | Yes |
| Sour flavor | 4.27 a | 3.97 ab | 3.66 b | 0.014 | Yes |
| Sweet taste | 3.18 b | 2.67 c | 3.76 a | < 0.0001 | Yes |
| Acidic taste | 3.90 b | 5.16 a | 3.31 b | < 0.0001 | Yes |
| Bitter taste | 4.04 ab | 4.35 a | 3.91 b | 0.033 | Yes |
| Hoppy flavor | 3.66 a | 3.83 a | 4.23 a | 0.230 | No |
| Malty flavor | 3.40 a | 3.29 a | 3.67 a | 0.161 | No |
| Fruity flavor | 5.26 a | 4.89 ab | 4.13 b | 0.051 | No |
| Perfume flavor | 2.68 a | 2.86 a | 2.69 a | 0.809 | No |
| Yeast flavor | 3.61 a | 3.89 a | 3.58 a | 0.411 | No |
| Alcohol flavor | 3.89 a | 3.74 a | 3.80 a | 0.842 | No |
| After taste | 5.37 a | 5.44 a | 4.71 b | 0.005 | Yes |
| Foaminess | 2.41 ab | 2.72 a | 2.06 b | 0.007 | Yes |
| Astringency | 3.72 b | 4.74 a | 3.45 b | < 0.0001 | Yes |

**Table S5.** Average sensory scores for all attributes evaluated in the descriptive analysis of the reference, *L. brevis* and *L. plantarum* beers. The table also includes sensory results from a commercial sour beer reference (Geuze). Beers receiving significantly different scores (p-value < 0.05, according to ANOVA analysis) are indicated with different letters, where group “a” has the highest score, group “b” second highest and “c” has the score concentration.

|  | Geuze | *L. plantarum* beer | *L. brevis* beer | *S. cerevisiae* | p-value | Significant |
| --- | --- | --- | --- | --- | --- | --- |
| Total intensity odour | 6.25 a | 5.46 ab | 5.30 b | 5.30 b | 0.012 | Yes |
| Sour odour | 3.29 b | 4.41 a | 3.45 b | 3.67 ab | 0.010 | Yes |
| Hoppy odour | 4.15 a | 4.01 a | 4.11 a | 4.08 a | 0.980 | No |
| Malty odour | 3.71 a | 3.71 a | 3.37 a | 3.40 a | 0.415 | No |
| Fruity odour | 3.25 b | 5.03 a | 3.63 b | 3.81 ab | 0.011 | Yes |
| Drie fruit odour | 3.34 a | 2.64 ab | 1.90 b | 1.59 b | 0.000 | Yes |
| Perfumed odour | 3.61 a | 3.18 ab | 2.29 b | 3.05 ab | 0.014 | Yes |
| Yeasty odour | 3.36 a | 3.42 a | 4.06 a | 3.69 a | 0.076 | No |
| Total intensity flavor | 6.91 a | 5.61 b | 6.05 b | 4.89 c | < 0.0001 | Yes |
| Sour flavor | 2.84 b | 4.19 a | 3.97 a | 3.66 a | < 0.0001 | Yes |
| Sweet taste | 2.67 c | 3.23 b | 2.67 c | 3.76 a | < 0.0001 | Yes |
| Acidic taste | 5.22 a | 3.92 b | 5.16 a | 3.31 b | < 0.0001 | Yes |
| Bitter taste | 6.17 a | 3.96 b | 4.35 b | 3.91 b | < 0.0001 | Yes |
| Hoppy flavor | 4.46 a | 3.69 a | 3.83 a | 4.23 a | 0.050 | Yes |
| Malty flavor | 3.34 a | 3.36 a | 3.29 a | 3.67 a | 0.191 | No |
| Fruity flavor | 3.23 b | 5.31 a | 4.89 a | 4.13 ab | 0.001 | Yes |
| Perfume flavor | 3.58 a | 2.54 b | 2.86 ab | 2.69 b | 0.015 | Yes |
| Yeast flavor | 3.43 a | 3.65 a | 3.89 a | 3.58 a | 0.299 | No |
| Alcohol flavor | 5.58 a | 3.88 b | 3.74 b | 3.80 b | < 0.0001 | Yes |
| After taste | 6.68 a | 5.36 b | 5.44 b | 4.71 c | < 0.0001 | Yes |
| Foaminess | 2.61 a | 2.31 ab | 2.72 a | 2.06 b | 0.006 | Yes |
| Astringency | 5.40 a | 3.61 b | 4.74 a | 3.45 b | < 0.0001 | Yes |
